# Localization of TFPI-2 in the nucleus modulates MMP-2 gene expression in breast cancer cells

**DOI:** 10.1038/s41598-017-14148-8

**Published:** 2017-10-19

**Authors:** Guangli Wang, Yao Zeng, Shaoying Chen, Deling Li, Wei Li, Yanchun Zhou, Robert H. Singer, Wei Gu

**Affiliations:** 1 Department of Pathophysiology, The Key Immunopathology Laboratory of Guangdong Province, Shantou University Medical College, Shantou, Guangdong Province 515041 China; 2 Anatomy and Structural Biology, Einstein College of Medicine, Bronx, New York, 10461 USA

## Abstract

TFPI-2 has recently been recognized as a tumor suppressor, which not only plays a fundamental role in modulation of ECM integrity, but also involves the regulation of many oncogenes. In this study, we investigated the potential mechanism of TFPI-2 in the suppression of breast cancer growth and invasion. We showed that, with either over-expression of TFPI-2 or after treatment with exogenous rTFPI-2, breast cancer cells exhibited reduced proliferation and invasion. We demonstrated that in addition to being secreted, TFPI-2 was also distributed throughout the cytoplasm and nucleus. Nuclear localization of TFPI-2 contributed to inhibition of *MMP-2* mRNA expression, which could be reversed after the nuclear localization signal was deleted. In the nucleus, interaction of TFPI-2 with Ap-2α attenuated the binding of AP-2α to the MMP-2 promoter, therefore reducing the transcriptional activity of the gene. Our results suggest that one of the mechanisms by which TFPI-2 inhibits breast cancer cell invasion could be via the regulation of MMP-2 gene transcription.

## Introduction

TFPI-2 is expressed and secreted into the extracellular matrix (ECM) of various types of human tissues, such as the liver, skeletal, muscle and pancreas^1–3^. Secretion of TFPI-2 into the ECM inhibits plasmin-mediated activation of MMPs and maintains the integrity of the ECM, therefore repressing tumor cell invasion^4,5^. It has been reported that in a number of tumor cell lines, exogenous application of recombinant TFPI-2 (rTFPI-2) in conditioned medium caused rTFPI-2 to be rapidly internalized by the cells and distributed in both the cytosolic and nuclear fractions^6^. Either constitutively expressed TFPI-2 or internalized TFPI-2 could be shuttled into the nucleus through an importin system^6^, suggesting that a secreted protein like TFPI-2 could associate with nuclear components and alter molecular events within the nucleus.

TFPI-2 has been recently recognized as a tumor suppressor, playing a suppressive role in tumor cell proliferation, apoptosis, invasion and angiogenesis^7–9^. Down-regulation of TFPI-2 expression, due to hypermethylation of its promoter, was seen in some highly aggressive tumors such as glioma, non-small cell lung cancer and breast cancers^8,10,11^. Decreased expression of TFPI-2 in cancer cells resulted in aberrant proliferation and invasion^1,7,12^. Immunohistochemical staining showed that the low levels of TFPI-2 were associated with breast cancer progression, recurrence and poor survival outcome after surgery^13^. TFPI-2 expression not only modulated ECM integrity, but also influenced the regulation of many oncogenes. For examples, reduced TFPI-2 levels were correlated with increased expression of *MMP-2* mRNA in pancreatic carcinomas and *MMP-1/MMP-3* mRNAs in lung cancer cells^14–16^. In the cytoplasm of HT1080 fibrosarcoma cells, TFPI-2 interacted with prosaposin to inhibit its invasion-promoting effects^17^.

In this study, we investigated the mechanisms underlying the invasion-suppressive effect of TFPI-2 in breast cancer cells. With over-expression of TFPI-2 or after treatment with exogenous rTFPI-2, we demonstrated that breast cancer cells exhibited a reduced ability to invade. In addition to being secreted, TFPI-2 was distributed both in the cytoplasm and nucleus. Translocation of TFPI-2 into the nucleus altered *MMP-2* mRNA expression through the interaction of TFPI-2 with AP-2α, a transcription factor important for the expression of many genes. Interaction of the two proteins attenuated the binding ability of AP-2α to the promoter of the *MMP-2* gene, thereby reducing its transcriptional activity. Our results revealed a biological activity of TFPI-2 in the nucleus, which suggests that one of the mechanisms that TFPI-2 suppresses migration and invasion of breast cancer cells could be mediated by the regulation of MMP-2 expression.

## Results

### Effects of TFPI-2 on proliferation and invasion of breast cancer cells

**Figure 1:**
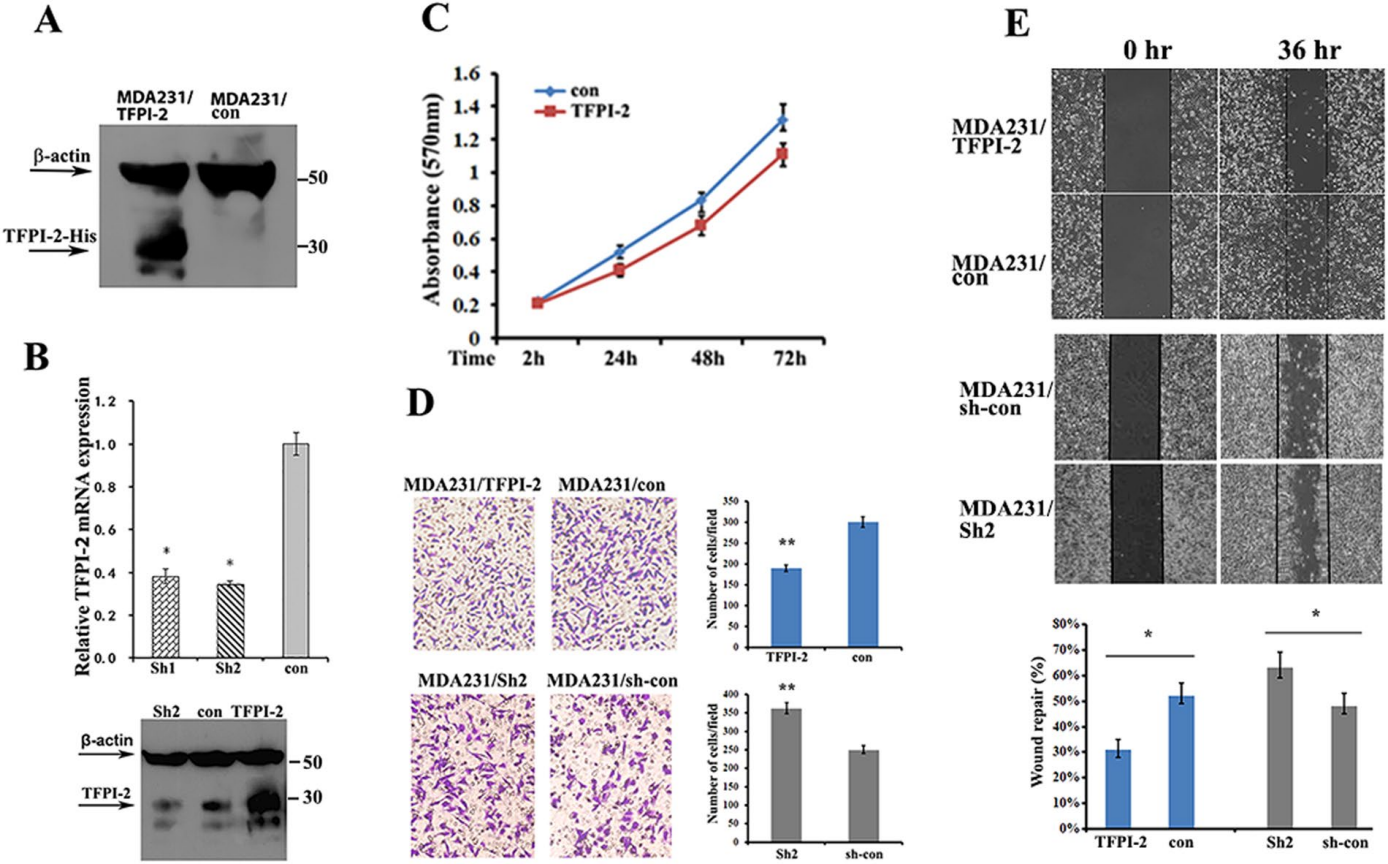
Overexpression of TFPI-2 in MDA231 cells suppresses cell invasion and migration. (**A**) Western blots showing the expression of TFPI-2 protein in MDA231/TFPI-2 and MDA231/con cells with an anti-His antibody. (**B**) Upper panel, expression of TFPI-2 mRNA was analyzed by qRT-PCR in TFPI-2 knockdown cells. *P < 0.05 as determined by one-way ANOVA followed by Tukey’s multiple comparison tests. Lower panel, MDA231 clones were subjected to western blots for the expression of TFPI-2 using antibodies against TFPI-2 and β–actin. (**C**) MTT assays indicate that overexpression of TFPI-2 decreased proliferation of MDA231 cells. Bars indicate standard error of mean from three independent experiments. P < 0.05 as determined by Student’s t-test. (**D**) Representative images and statistical analyses of transwell assays indicate the invasive capacity of MDA/TFPI-2 vs. MDA231/con cells and MDA231/Sh2 vs. MDA231/sh-con cells. **P < 0.01 as determined by Student’s t-test. (**E**) Representative micrographs of wound healing assay of the indicated cells. Wound closures were photographed at 0 and 36 hours after wounding. Statistical analysis was assessed from three independent experiments; ± SD. *P < 0.05 as determined by Student’s t-test.

To study the capability of TFPI-2 in suppression of the proliferation and invasiveness of breast cancer cells, we established three MDA231 cell lines: one was infected with a lentivirus vector expressing His-tagged human TFPI-2 (MDA231/TFPI-2), and the other two were TFPI-2 down-regulated cell clones using shRNA approaches (MDA231/Sh1 and MDA231/Sh2). Constitutive expression of TFPI-2 was verified by western blots, in which characteristic bands of TFPI-2 were shown in contrast to the MDA231 control cells that were infected with an empty vector (MDA231/con, Fig. 1A). Knockdowns of TFPI-2 were confirmed by qRT-PCR and western blots (Fig. 1B, Supp. Figure 1A). Since Sh1 and Sh2 cells showed similar cell proliferation and invasion capability (Supp. Figure 1B), hereafter we used the Sh2 cell line for most of the experiments. MTT assays indicated that knockdown of TFPI-2 expression increased, while overexpression of TFPI-2 decreased the capability of cell proliferation (Fig. 1C, Supp. Figure 1B). Transwell assays showed that cell invasion was markedly decreased in MDA231/TFPI-2 cells, and was increased in MDA231/Sh2 cells compared to control and parental cells (Fig. 1D and Supp. Figure 1C). However, increased ability of cell invasion in MDA231/Sh2 cells could be rescued when TFPI-2 was re-expressed (Supp. Figure 1D). Furthermore, wound healing analyses showed that the wound repair was delayed in MDA231/TFPI-2 cells and was enhanced in cells when TFPI-2 was down-regulated (Fig. 1E). Delayed wound closure in TFPI-2-expressing cells and control cells was also observed in the presence of mitomycin-C (10 µM) that inhibits cell proliferation (Supp. Figure 1E and 1F). These results indicate a suppressive role of TFPI-2 on invasiveness and migration of breast cancer cells.

### Stimulation of cultured MCF7 cells with TFPI-2 inhibits proliferation and invasion

**Figure 2:**
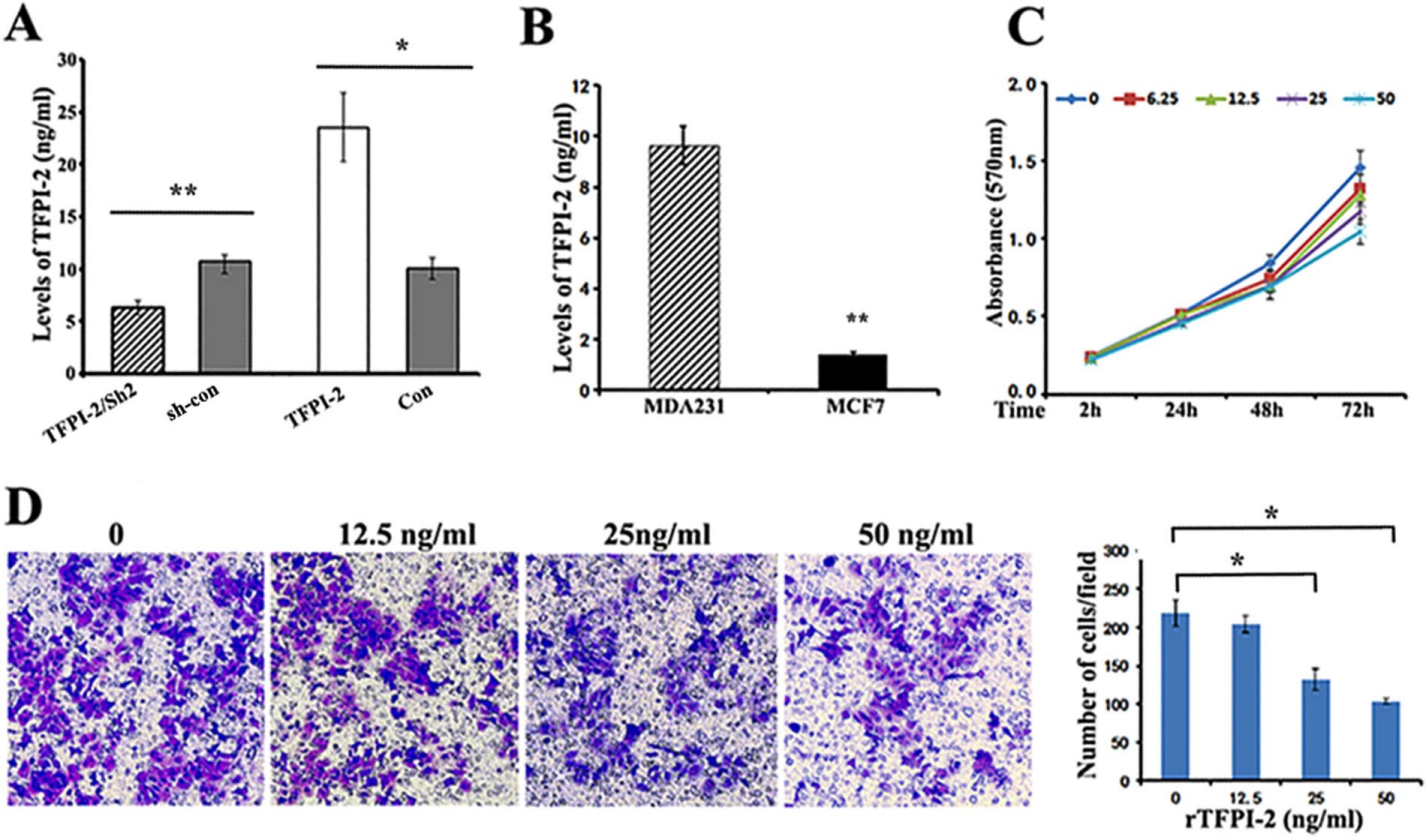
Stimulation of recombinant TFPI-2 (rTFPI-2) inhibits proliferation and invasion of breast cancer cells. (**A**) ELISA assays indicate the relative levels of secreted TFPI-2 in the culture medium of stable MDA231 cell lines. Data were analyzed from three independent experiments.*P < 0.05 as determined by Student’s t-test. (**B**) ELISA assays show that the levels of TFPI-2 in culture medium of MDA231 cells were higher than that in MCF7 cells (~6.85 fold). Data were analyzed from three independent experiments. **P < 0.01 as determined by Student’s t-test. (**C**) Effect of exogenously offered rTFPI-2 on proliferation of MCF7 cells. MCF7 cells were cultured in the medium containing various concentrations of rTFPI-2 (0, 6.25, 12.5, 25, 50ng/ml). Cell viability was measured by MTT assays at the time of 2, 24, 48, and 72hrs. P < 0.05. (**D**) Transwell assays show the invasive capacity of MCF7 cells after incubation with exogenous rTFPI-2 (0, 12.5, 25, 50ng/ml). Representative images of the assays were shown and statistical data were analyzed from three independent experiments. *P < 0.05 as determined by one-way ANOVA followed by Tukey’s multiple comparison tests.

A number of studies have reported that TFPI-2 is produced and secreted into the extracellular medium where it acts as a serine proteinase inhibitor^7,9,13^. To determine the secretion levels of TFPI-2, we employed ELISA experiments. In three MDA231 stable cell lines, levels of secreted TFPI-2 were 2.32 fold-higher in MDA231/TFPI-2 cells and 0.75 fold-lower in MDA231/Sh2 cells than that in the control cells (Fig. 2A). Examination of the extracellular levels of TFPI-2 in different breast cancer cell lines indicated that MDA231 cells expressed relatively higher levels of TFPI-2 than MCF7 and T47D cells (Fig. 2B and Supp. Figure 2).

In order to investigate the effect of extracellular TFPI-2 on cell invasion, we stimulated MCF7 cells with culture medium containing increasing concentrations of human recombinant TFPI-2 (rTFPI-2, 0 ng/ml to 50 ng/ml) and cultured for 72 hrs. MTT assays showed that the rate of proliferation was sequentially reduced in the cells that were treated with increasing amount of rTFPI-2, compared to the cells without the rTFPI-2 treatment (Fig. 2C). Transwell assays also showed decreased invasive capability of MCF7 cells when cultured in medium containing rTFPI-2 (Fig. 2D). Since MDA231/TFPI-2 cells secreted relatively high levels of TFPI-2, we next treated MCF7 cells with the conditioned culture medium harvested from MDA231/TFPI-2 cells. Consistent with the prior experiments, proliferation and invasiveness of MCF7 cells were also considerably reduced in the culture medium containing higher levels of TFPI-2 (Supp. Figure 3). These results demonstrated that exogenous application of TFPI-2 inhibits breast cancer cell proliferation and invasion.

### TFPI-2 lowers the expression of matrix metalloproteinase2 (MMP-2) mRNA in MDA231 cells

**Figure 3:**
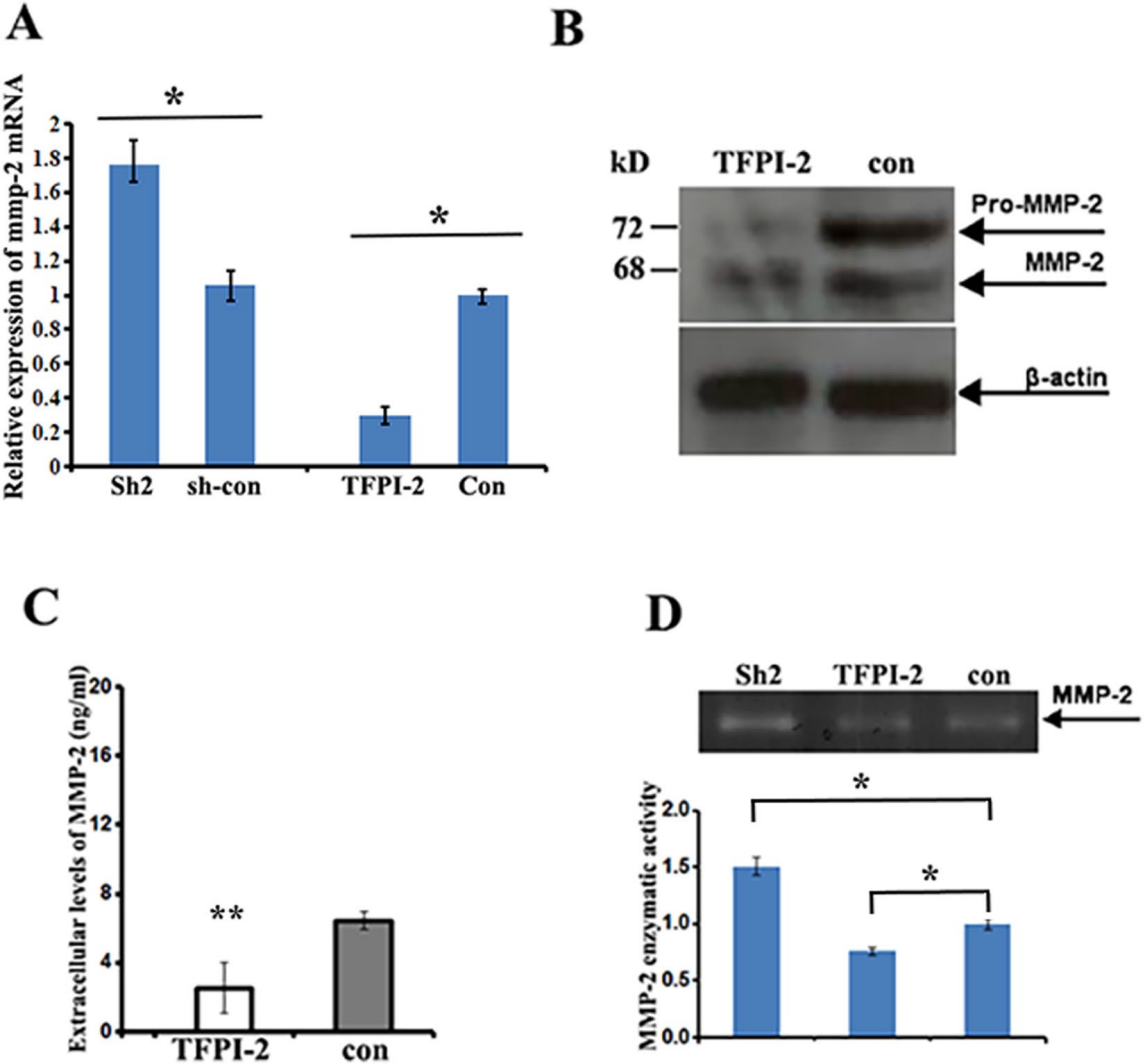
Down-regulation of MMP-2 expression in MDA231/TFPI-2 cells. (**A**) qRT-PCR was performed to analyze the relative levels of MMP-2 mRNA in MDA/TFPI-2 vs. MDA231/con cells and MDA231/Sh2 vs. MDA231/sh-con cells. Bars indicate the standard error of mean from three independent experiments, *P < 0.05 as determined by Student’s t-test. (**B**) Western blots indicate the expression of MMP-2 protein in MDA231/TFPI-2 and MDA231/con cells. (**C**) ELISA assays were used to measure the secreted levels of MMP-2 protein in culture medium of MDA231 cell clones. (**D**) Upper panel, a representative gelatin zymography showing the enzymatic activity of MMP-2 in the culture medium of MDA231 cell lines. Lower panel, the intensity of the bands were assessed by densitometric semi-quantitation from three independent experiments and depicted by a bar diagram, ± SD. *P < 0.05 as determined by one-way ANOVA followed by Tukey’s multiple comparison tests.

Higher expression of metalloproteinases (MMPs) has been shown in breast cancer cells, and a correlation of reduced TFPI-2 levels with increased expression of MMP-2 mRNA was reported in pancreatic cancers^14,18^. Using qRT-PCR to examine the correlation of TFPI-2 with the expression of *MMP-2*, *MMP-1* and *MMP-9* mRNAs in MDA231 cells, we found that the levels of MMP-2 mRNA were significantly decreased in MDA231/TFPI-2 cells and were increased in TFPI-2 knockdown cells (Fig. 3A). Reduced MMP-2 mRNA expression was also detected in the T47D/TFPI-2 cells in which the exogenous TFPI-2 was constitutively expressed (Supp. Figure 4A and 4B). However, relative levels of *MMP-1* mRNA and *MMP-9* mRNA were barely changed in the cell clones examined (Supp. Figure 4C and D). As a result, cellular MMP-2 protein levels (Fig. 3B), secreted levels of MMP-2 in culture medium (Fig. 3C) and the enzymatic activity of MMP-2 (Fig. 3D) were lower in the cells constitutively expressing TFPI-2. These experiments suggest that TFPI-2 decreases MMP-2 mRNA expression and subsequently the protein’s activity in breast cancer cells.

### Translocation of TFPI-2 into nucleus affects the promoter activity of the MMP-2 gene

**Figure 4:**
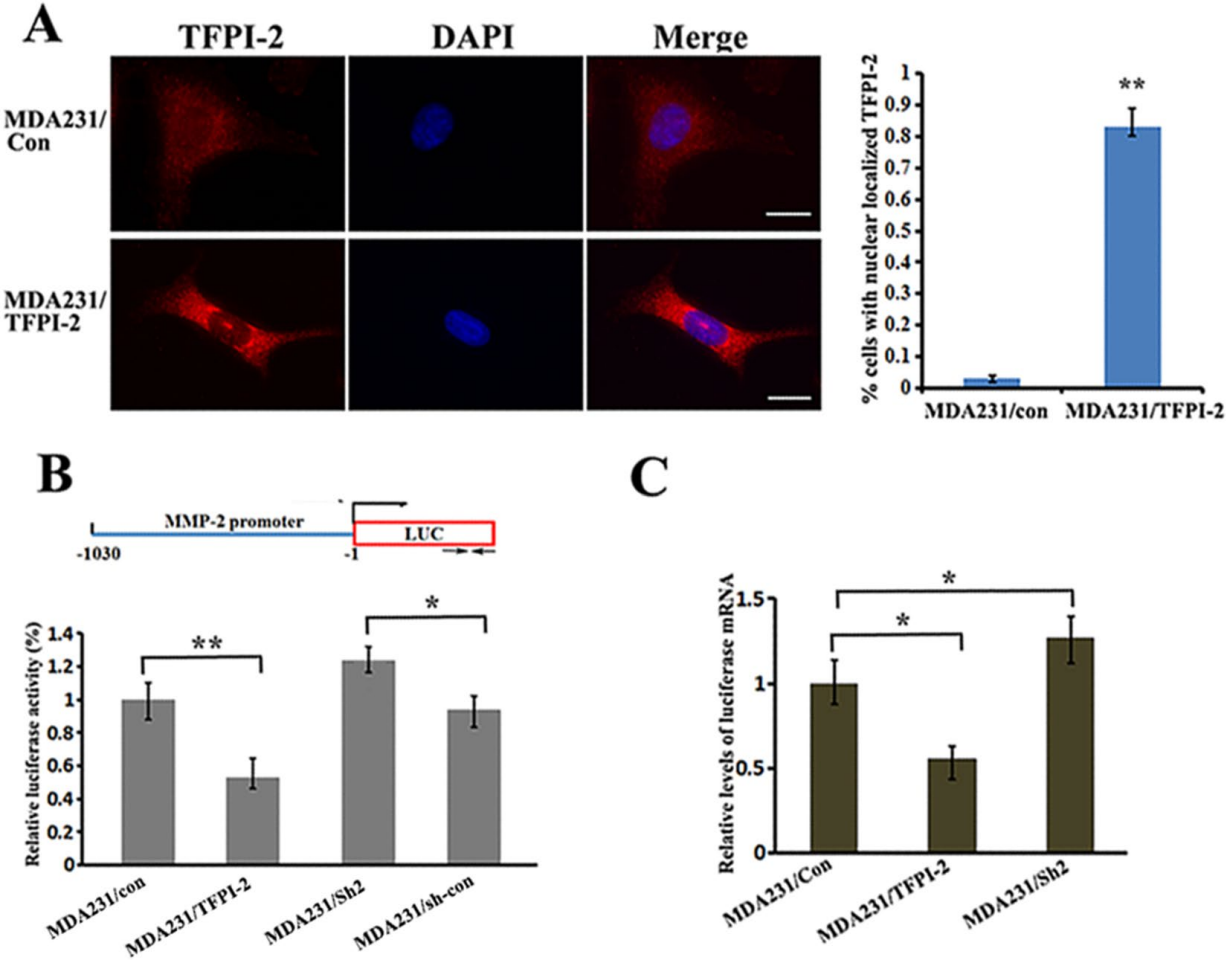
Translocation of TFPI-2 into nucleus decreased promoter activity of the MMP-2 gene. (**A**) Representative images show the cellular distribution of TFPI-2 in MDA231 cell lines (left). Scale bars: 10 μm. An average of 100 TFPI-2 overexpression cells was examined and the percentage of the cells with enriched TFPI-2 protein in the nucleus is presented (right). **P < 0.01 as determined by Student’s t-test. (**B**) Analyses of MMP-2 promoter activity in MDA231 cell clones. Upper: schematic representation of the luciferase reporter driven by the MMP-2 promoter. Arrows indicate the transcription start site for the luciferase gene. Lower: Luciferase reporter driven by the MMP-2-promoter and an internal control Renilla luciferase plasmid were co-transfected into cultured MDA231 cell lines. The firefly luciferase activity was normalized to the Renilla luciferase activity and the relative luciferase activity is represented. The results were the average of a total of three independent experiments each carried out in triplicate. *P < 0.05 as determined by Student’s t-test. (**C**) Levels of reporter firefly luciferase mRNA were determined by RT-PCR and normalized to Renilla luciferase mRNA for each cell line. Mean values (+SD) from three independent experiments are shown. *P < 0.05, **P < 0.01 as determined by one-way ANOVA followed by Tukey’s multiple comparison tests.

Accumulating evidence suggests that many proteins could play distinctive roles depending upon their cellular location^19,20^. Using immunofluorescence assays to examine the intracellular localization of TFPI-2, we detected that TFPI-2 was distributed both in the cytoplasm and the nucleus (Fig. 4A, upper panels). Interestingly, overexpression of TFPI-2 greatly enhanced its nuclear translocation (Fig. 4A, lower panels). Enriched nuclear localization of TFPI-2 could be seen in over 80% of the cells (Fig. 4A, right). We hypothesized that localization of TFPI-2 inside the nucleus could affect the transcription of the MMP-2 gene. To address this, we constructed a firefly luciferase reporter (pGL2) driven by the MMP-2 promoter (–1030/-1, refer to Fig. 4B, upper). The constructs were transiently co-transfected with a renilla reporter driven by a SV-40 promoter into MDA-231/TFPI-2, MDA231/Sh2 and MDA-231/con cells. After 24 hrs of culture, relative luciferase activity was significantly reduced in cells expressing TFPI-2, while the activity was increased by 25% in TFPI-2 knockdown cells in compared to that in the control cells (Fig. 4B, lower). Decreased luciferase activity was also detected when the luciferase reporter constructs were transfected into T47D/TFPI-2 cells (Supp. Figure 5A). To determine whether changes of luciferase activity in transfected cells were due to decreased transcription of the reporter, we performed qRT-PCR assays and found that the mRNA levels of the luciferase reporter in MDA231/TFPI-2 cells was lower (Fig. 4C). Thus, the reduction of *MMP-2* gene expression could result from TFPI-2 overexpression and nuclear location.

### Blocking the import of TFPI-2 into nucleus rescues the promoter activity of the MMP-2 gene

**Figure 5:**
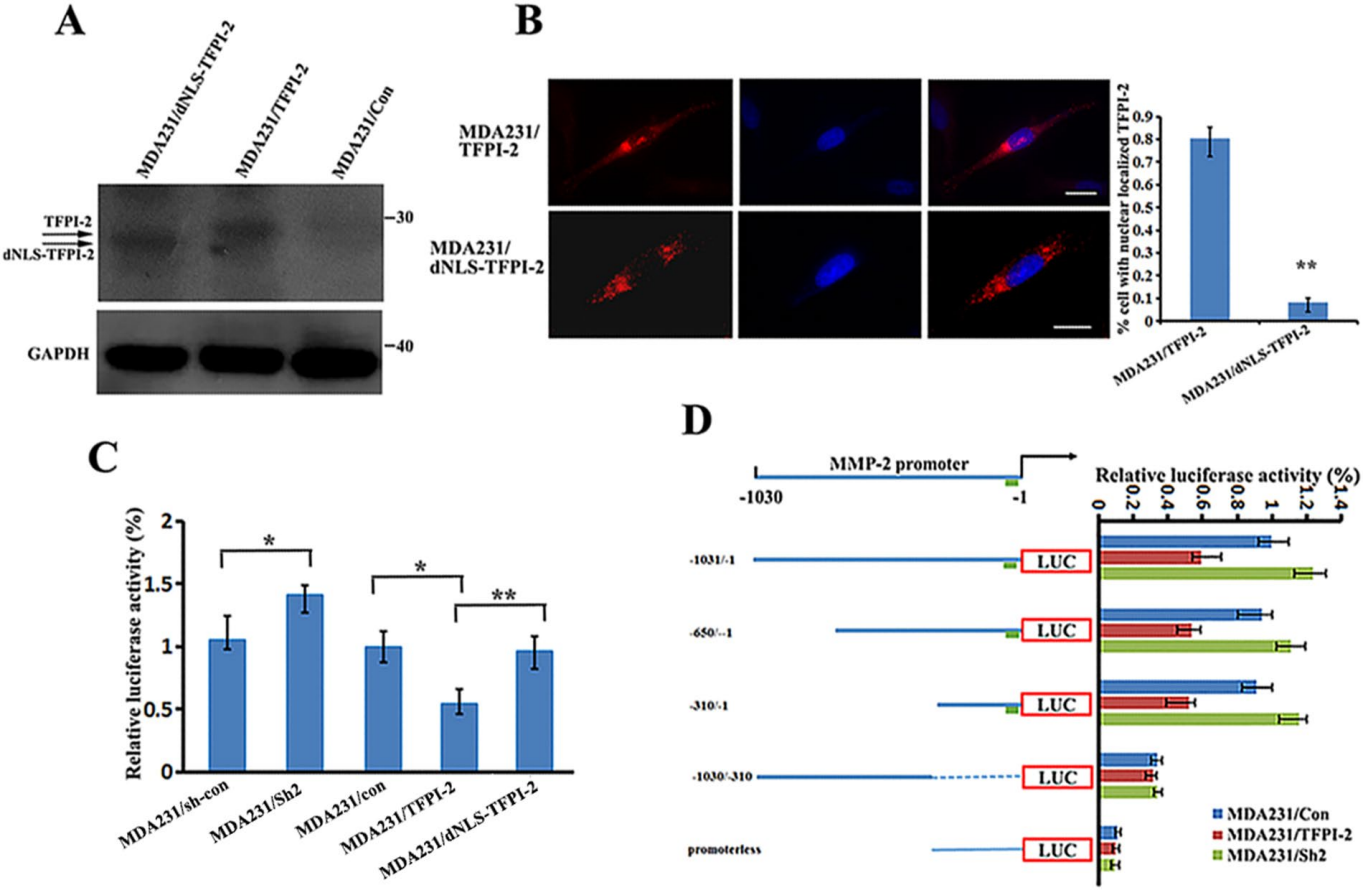
The active promoter region of the MPP-2 gene contains the binding site for AP-2α that interacts with TFPI-2. (**A**) Western blots show the expression of TFPI-2 and dNLS-TFPI-2 in MDA231 stable cell lines. GAPDH was used as a loading control. (**B**) Representative immunofluorescent assays indicate that deletion of the NLS blocked the nuclear localization of TFPI-2 in dNLS-TFPI-2 cell line. Scale bars: 10 μm. An average of 100 cells of each cell type was examined. **P < 0.01 as determined by Student’s t-test. (**C**) Analyses of the effect of nuclear localized TFPI-2 on MMP-2 promoter activity. Luciferase reporter driven by the -1030 to -1 bp of the MMP-2 promoter and an internal control Renilla luciferase plasmid were co-transfected into stable MDA231 cell lines. The firefly luciferase activity is normalized to the Renilla luciferase activity and the relative luciferase activity is represented. The results are the average of a total of three independent experiments each carried out in triplicate. *P < 0.05, **P < 0.01 as determined by Student’s t-test and by one-way ANOVA followed by Tukey’s multiple comparison tests. (**D**) Analyses of active regions of the MMP-2 promoter. Left: schematic representation of the luciferase reporter constructs driven by 5′ nested deletions of the MMP-2 promoter. Relative positions of the promoter region in each construct are marked. The arrow indicates the putative transcription initiation sites for the MMP-2 gene. The green block indicates the potential binding element for AP-2, SP1 and PEA3. Right: MMP-2 promoter constructs and an internal control Renilla luciferase plasmid were co-transfected into cultured MDA231/TFPI-2 cells. The firefly luciferase activity from each construct was normalized to the Renilla luciferase activity and the relative luciferase activity is represented as a percentage of the MMP-2 promoter construct (-1030/-1). The results are the average of three independent experiments each carried out in triplicate, ± SD. P < 0.01 as determined by one-way ANOVA followed by Tukey’s multiple comparison tests.

TFPI-2 contains a nuclear localization signal (NLS) that is responsible for the protein translocation into the nucleus^6^. To address the direct effect of nuclear TFPI-2 on *MMP-2* transcription, we established a MDA231 cell line (MDA231/dNLS-TFPI-2) in which a truncated NLS-lacking TFPI-2 was expressed (Fig. 5A). Immunostaining analysis indicated that TFPI-2 without its NLS was mostly located in the cytoplasm and the nuclear signal of the protein was greatly decreased (Fig. 5B). We then transfected the luciferase reports into MDA231/con, MDA231/Sh2, MDA-231/TFPI-2 and MDA231/dNLS-TFPI-2 cells. We found that the relative luciferase activity was very similar between control cells and the cells expressing NLS-free TFPI-2. However, in compared to the cells that expressed full-length TFPI-2, luciferase activity in the NLS-free TFPI-2 cells was increased about 50% (Fig. 5C), indicating that blocking translocation of TFPI-2 into the nucleus enhanced the promoter activity of the MMP-2 gene.

To identify the promoter region that is responsible for *MMP-2* transcription, we generated luciferase reporters under the control of various lengths of the 5′ flanking region of the MMP-2 promoter (Fig. 5D). A promoterless parent pGL2 vector (pGL2-Basic) was used as a negative control for luciferase activity (Fig. 5D, left lower). After transient co-transfection with a Renilla luciferase plasmid into cultured MDA231/TFPI-2 cells, firefly luciferase activities were determined for each transfection and normalized to corresponding renilla luciferase activity, respectively. While the luciferase activity of the promoterless vector was barely detected, luciferase reporters driven by various lengths of the *MMP-2* promoter were relatively active in the cells. Sequential deletion of the *MMP-2* promoter from -1030 to -300 bp did not significantly change the reporter activity, which suggested that the -300 bp flanking region was sufficient for *MMP-2* promoter activity (Fig. 5D, right). The 300 bp promoter region of the MMP-2 gene contained few putative elements for transcription factor binding, including Ap2 (-61 bp), Sp1 (-70 bp and -91bp), PEA3 (-145 bp) and CREB (-300 bp). In these elements, the AP2 and SP1 (-91) sites have been reported to contribute to most of the promoter activity^21^.

### Interaction of TFPI-2 with AP-2α attenuates the binding capability of AP-2α on the MMP2 gene promoter

**Figure 6:**
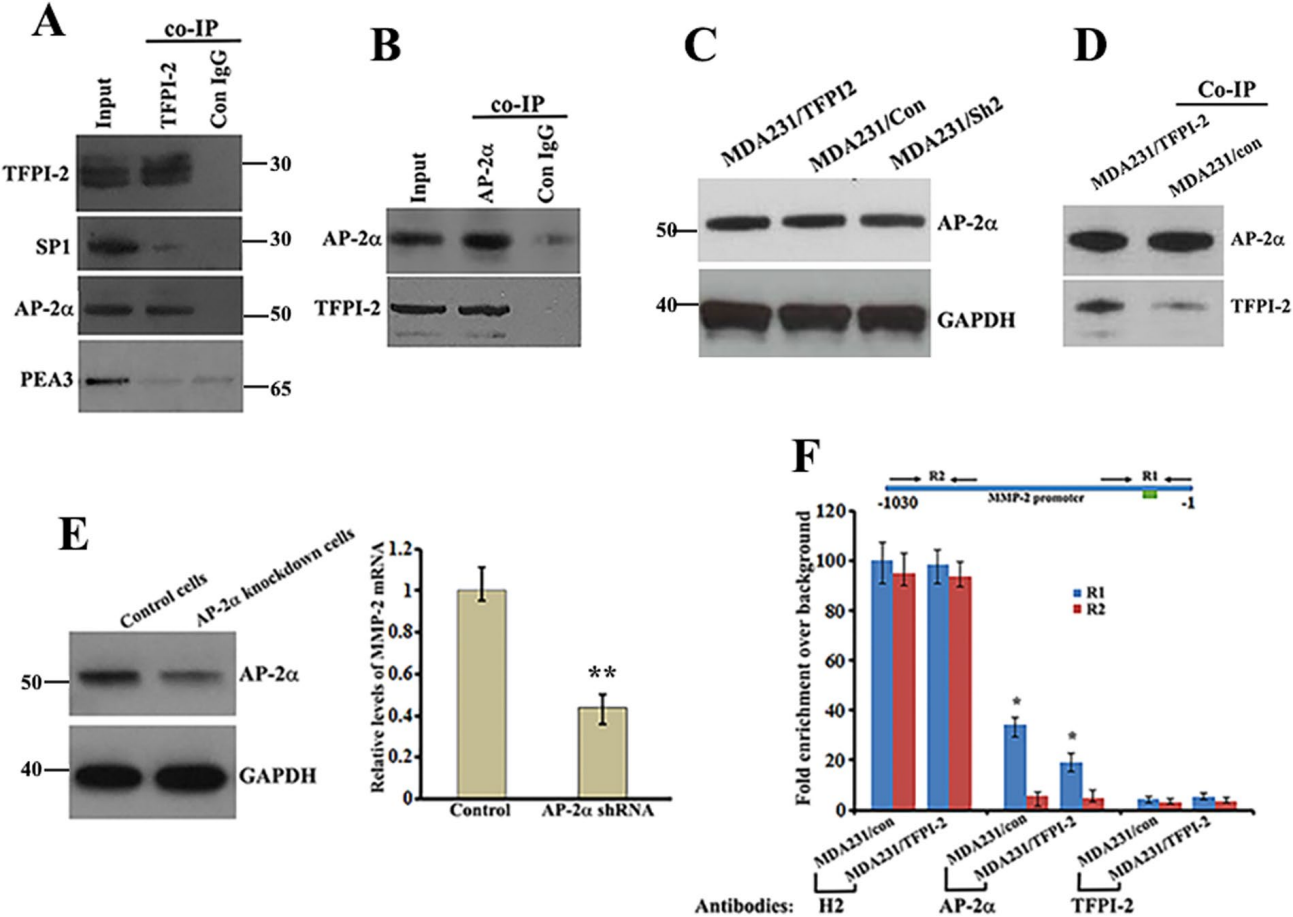
Interaction of TFPI-2 with AP-2α reduces the transcriptional activity of the MMP2 promoter. (**A**) Protein A beads conjugated with TFPI-2 antibodies or normal IgG were used for Co-IP experiments to analyze the interaction of TFPI-2 with putative transcriptional factors in the nuclear extracts of MDA231/TFPI-2 cells. The precipitates were subjected to western blots probing by anti-TFPI-2, anti-PEA3, anti-AP-2α and anti-SP1 antibodies. The samples are derived from the same experiment and that blots were processed in parallel. (**B**) Protein A beads conjugated with AP-2α antibodies or normal IgG were used for the reciprocal Co-IP experiments to detect the association of AP-2α with TFPI-2 in the nuclear extracts. The precipitates were subjected to western blot analyses. (**C**) Western blots show the levels of AP-2α in MDA231 cell lines with or without TFPI-2 overexpression. GAPDH was used as a loading control. (**D**) Co-IP using AP-2α antibodies confirmed association of TFPI-2 with AP-2α (**E**) Left: western blots show the down-regulation of AP-2α in MDA231 cells. Right: qRT-PCR indicates that knockdown of AP-2α decreases the expression of MMP-2 mRNA in MDA231 cells. ** < 0.01 as determined by Student’s t-test. (**F**) ChIP assays were performed to detect the binding ability of AP-2α to the MMP2 gene promoter. Upper panel: schematic presentation of the MMP-2 gene promoter and the two regions to be analyzed by qPCR. The R1 region contains the putative binding sites for AP-2α. Arrows represent primers used for qPCR analysis. Amplicon lengths for R1 and R2 are about 300 bp, respectively. Lower panel: Protein A beads conjugated with Histone 2 (H2), AP-2α or TFPI-2 antibodies were used for ChIP assays. After immunoprecipitation, amplicons corresponding to R1 and R2 were amplified and analyzed. Relative levels of the amplicons are statistically analyzed and the data is presented as means ± SD from three independent experiments. *P < 0.05 as determined by Student’s t-test.

To determine whether TFPI-2 could interact with transcription factors, we prepared nuclear extracts for MDA231/TFPI-2 cells and performed Co-IP experiments using TFPI-2 antibodies. Anti-TFPI-2 antibody was able to co-precipitate AP-2α, but not SP1 or PEA3 (Fig. 6A). A reciprocal experiment using AP-2α antibody confirmed the *in vivo* interaction of AP-2α with TFPI-2 (Fig. 6B). Interestingly, formation of AP-2α/TFPI-2 complex did not influence the stability of AP-2α, since the levels of AP-2α were not changed when TFPI-2 was overexpressed (Fig. 6C). Using AP-2α antibodies to Co-IP TFPI-2 in MDA231/TFPI-2 and MDA231/con cell lines further indicated that TFPI-2 was indeed associated with AP-2α (Fig. 6D). We then introduced a lentiviral vector expressing specific AP-2α shRNAs into MDA231 cells and obtained a pronounced knockdown of AP-2α protein levels (Fig. 6E, left). In AP-2α knockdown cells, both MMP-2 mRNA expression (Fig. 6E, right) and the invasive potential of the cells (Supp. Figure 5B) were reduced. Moreover, over-expression of MMP-2 in MDA231/TFPI-2 cells resumed the invasive phenotype (Supp. Figure 6). These results suggest the importance of TFPI-2/AP-2α interaction on transcription of the MMP-2 gene.

We hypothesized that nuclear interaction of TFPI-2/AP-2α could affect the binding activity of the AP-2α to the *MMP-2* promoter, thus decreasing the transcriptional activity of the gene. To address this, we carried out ChIP assays, in which chromatin associated AP-2α was immunoprecipitated, and enrichment of the specific promoter regions of the *MMP-2* was measured by qPCR. Two specific amplicons (R1, -300/ + 1 and R2, -1030/-650) of the MMP-2 promoter were analyzed (Fig. 6F, upper panel). The experiments showed that the two amplicons were highly enriched after the ChIPs of Histone 2 antibodies. The R1 amplicon, but not the R2 amplicon, was enriched in MDA231/con cells after AP-2α ChIPs. However, when TFPI-2 was constitutively expressed, the enrichment was significantly decreased. No enrichment of the R1 amplicon was identified in the ChIPs of TFPI-2 antibodies, eliminating the possibility that TFPI-2 could directly bind to the promoter (Fig. 6F, lower panel). Effect of TFPI-2 on the binding of AP-2α to the MMP-2 promoter was further confirmed by a combination assay of co-IP and PCR experiments (Suppl. Figure 6). These results indicated that the interaction of TFPI-2 with AP-2α could reduce the binding ability of AP-2α to the *MPP-2* gene promoter, resulting in decreased expression of the *MMP-2* gene.

## Discussion

Differences in the cellular localization of a protein can generate multiple functions and allow a single protein to control different specific biological outcomes^20,22,23^. TFPI-2 is a member of the Kunitz-type serine proteinase inhibitor family and has been shown to protect the extracellular matrix from degradation, thereby inhibiting tumor cell invasion and metastasis^4,9,24^. The expression of TFPI-2 in tumors is inversely related to the degree of malignancy. While investigating the biological function of TFPI-2 in breast cancer cells, we observed that breast cancer cells, either constitutively expressing TFPI-2 or stimulated with rTFPI-2, have decreased invasive ability that could be reversed by TFPI-2 down-regulation. More interestingly, we found that TFPI-2 is not only secreted, but also generally expressed in the cytoplasm and nucleus. Nuclear localization of TFPI-2 allowed the protein to interact with the transcription factor, AP-2α, and regulates MMP-2 gene expression. Our studies revealed a novel function that TFPI-2, in addition to maintaining the ECM integrity, plays an important role in regulating gene expression.

Lower TFPI-2 expression is associated with breast cancer progression, recurrence and poor survival^13^. In a variety of tissue cells, secretion of TFPI-2 into the extracellular matrix (ECM) represses the plasmin-mediated activation of matrix pro-metalloproteinases and could thus regulate ECM integrity^15,25^. A number of studies have indicated that secreted proteins can also localize in the cytoplasm and nucleus to perform distinct functions^19,26^. For example, NAG-1/GDF15, when present inside the nucleus, alters gene expression and interferes with the TGF-β1-induced Smad signaling pathway^20^. Secreted proteins such as the basic fibroblast growth factor and the odontogenic ameloblast associated protein have been reported to be expressed in the ECM, as well as the nucleus and cytoplasm^27^. In addition to breast cancer cells, localization of TFPI-2 in the cytoplasm and the nucleus has also been reported previously in HT-1080 and other cell lines^6,17^. Interestingly, in the cytoplasm of human HT1080 fibrosarcoma cells, TFPI-2 was shown to interact with PSAP (prosaposin), and this interaction inhibited the invasion-promoting effects of PSAP^17^. In our study, we demonstrated that the nuclear localization of TFPI-2 was able to regulate *MMP-2* gene transcription. This regulation was based on the molecular interaction of TFPI-2 with the transcription factor AP-2α, which bound to the regulatory region of the MMP-2 gene promoter. The TFPI-2/AP-2α interaction caused the reduction of the binding ability of AP-2α to the MMP-2 promoter, thus decreasing the transcription activity of the MMP-2 gene and inhibiting cell invasion. Our studies indicate that the different cellular localizations of TFPI-2 produce multiple functions via interactions with different protein targets, leading to the overall inhibition of cell invasiveness.

Many serine protease inhibitors including TFPI-2 accumulate in cell nucleus^28–31^. However, the biological role of the nuclear-associated serine protease inhibitors remained to be identified. Our studies discovered a potential function for TFPI-2 in regulation of MMP-2 gene expression. This regulation is not due to the nature of TFPI-2 as a protease inhibitor, but rather due to its interaction with AP-2α, leading to diminished binding ability of AP-2α to the *MMP-2* promoter. This result was different from a previous report showing that nuclear localized cysteinyl proteinases caused proteolysis of transcription factors in differentiated embryonal carcinoma cells^32^. The molecular mechanism of how the TFPI-2/AP-2α interaction results in AP-2α disassociation from the MMP-2 promoter is currently under investigation.

AP-2 proteins are a family of developmentally-regulated transcription factors. They are encoded by five different genes (alpha, beta, gamma, delta, and epsilon) and share a common structure. Down regulation of AP-2α in MCF-7 tumor cells resulted in a significant reduction of chemotherapy-induced apoptosis, migration, and motility and an increase in adhesion^33^. We have also shown a role for AP-2α in regulating MMP-2 gene transcription and promoting invasion of MDA231 cells. Since the AP-2 family plays relevant roles in cell growth, differentiation, and adhesion by controlling the transcription of target genes, we hypothesize that in addition to *MMP-2*, interaction of TFPI-2 with AP-2α could also regulate the transcription of other genes.

In conclusion, our study has elucidated a novel biological mechanism by which TFPI-2 suppresses breast cancer cell proliferation and invasion. We demonstrated that the translocation of TFPI-2 into the cell nuclei allowed the protein to interact with AP-2α and modulate the transcription of the MMP-2 gene. Our study provides initial evidence that, in addition to maintaining the integrity of ECM, TFPI-2 suppresses breast cancer cell invasion through the regulation of *MMP-2* gene expression.

## Methods

### Enzymes, antibodies and chemicals

Restriction endonucleases were purchased from New England Biolabs, Inc. (Ipswich, MA, USA). Rabbit anti-TFPI-2, goat anti-TFPI-2 and mouse anti-AP-2α antibodies were purchased from Santa Cruz Biotechnology, Inc. (Dallas, USA). Mouse anti-TFPI-2 antibody was purchased from R&D systems (Minneapolis, MN, USA). Mouse anti-MMP2 antibody was from Novus Biologicals (Littleton, CO, USA). Anti-β-actin, anti-tubulin-α and anti-lamin B antibodies were purchased from Bios Inc. (Tokyo, Japan). Goat anti-rabbit and goat anti-mouse antibodies were purchased from Sigma-Aldrich Chemical Co (St. Louis, MO, USA). Cy3-conjuagted donkey anti-rabbit, anti-mouse and anti-goat antibodies, FITC-conjugated donkey anti-goat and anti-rabbit antibodies were purchased from Jackson Immunoresearch Laboratories (West Grove, PA, USA).

### Cell culture

Human breast cancer cell lines MDA-MB-231 (MDA231), MCF7, and T47D cells and HEK293T cells were grown in DMEM supplemented with 10% fetal bovine serum (FBS), penicillin (100 units/ml) and streptomycin (100 μg/ml) in a humidified atmosphere containing 5% CO_2_ at 37 °C. Cells in the logarithmic growth phase were collected for experiments.

### Vector construction, cell transfection and infection

A Full-length TFPI-2 construct (1–233 aa) including a C-terminal His_6_-tag and a truncated TFPI-2 construct (1–192 aa) without the NLS (nuclear localization signal) were PCR amplified and subcloned into a lentiviral expression vector (pCIP2). pGIPZ lentiviral vectors encoding shRNA for TFPI-2 knockdown were constructed. The sequences of shRNA are listed in Supplemental Table 1. Cell transfection and infection with lentiviral vectors were performed as previously described^34^. Infected cells were cultured and selected with puromycin (2–4 μg/ml) for 2–3 weeks. Expression of TFPI-2, dNLS-TFPI-2, and AP-2α in selected cells was verified by RT-PCR or Western blots.

### Total RNA extraction and reverse transcription

Total RNA was extracted from MDA231, MCF7 and T47D cells using an RNeasy Mini Kit purchased from Qiagen Inc, (Valencia, California, USA) according to the manufacturer’s instructions. RNA was quantified using the Nanodrop ND-2000c Spectrophotometer (Thermo Fisher Scientific, MA, USA). Collected RNA was used immediately or stored at −80 °C. For cDNA synthesis, 1 μg of total RNA was reverse-transcribed into cDNA using oligo dT and the Superscript Amplification System (Invitrogen Inc, USA).

### Quantitative Real time PCR (qRT-PCR)

Quantitative RT-PCR was carried out using an ABI 7500 Fast real time PCR system (Thermo Fisher Scientific, MA, USA) and the SYBR Green PCR Master Mix (Qiagen Inc). Specific primers used for qPCR were listed in Supplemental Table 2. Cycling conditions were as follows: 50 °C for 2 min, 95 °C for 15 min followed by 30 cycles of 95 °C for 15 sec and 60 °C for 30 sec. For quantification, the target genes were normalized to the internal standard GAPDH gene. Relative expression levels were calculated using comparative ^ΔΔ^Ct method.

### Cell extracts preparation and Western blot analysis

To prepare total cell lysates, cells were rinsed with ice-cold PBS and lysed in a buffer containing 50 mm HEPES, 150 mm NaCl, 50 mm NaF, 1% Triton X-100, 10% glycerol, 5 mm EDTA and protease inhibitor cocktail (Roche, Basel, Switzerland). Supernatant was recovered after the lysate was centrifuged for 15 min at 12,000 × g at 4 °C. Reagents for making nuclear and cytosolic fractions were purchased from Yeasen Inc (Shanghai, China). Protein samples were separated by 4–12% SDS-PAGE (Invitrogen Inc, USA) and transferred onto 0.45 μm nitrocellulose membranes. Following one hour incubation in 5% fat-free milk, the membranes were probed with selected primary antibodies overnight at 4 °C. Blots were then washed, incubated for one hour with respective secondary antibodies, and visualized using enhanced chemiluminescence reagents (Amersham Inc). For sequential immunoblotting experiments, the blots were washed with Tris-buffered saline, treated with Restore Western Blot Stripping Buffer (Thermo Scientific, Rockford, IL, USA), washed and re-blocked, and incubated with primary antibodies if necessary.

### Enzyme-linked immunosorbent assay (ELISA)

ELISA kits for detecting TFPI-2 and MMP-2 proteins were purchased from (CUSABIO Inc, Wuhan, China). 1 × 10^6^ cells were seeded in 6-well tissue culture plates and cultured with serum-free medium for 24 hrs. Culture medium was collected, and centrifuged at 12,000 × g for 10 min to remove debris. Levels of TFPI-2 and MMP-2 proteins in the culture medium were analyzed by the ELISA kits according to the manufacturer’s instructions.

### Cell proliferation assay

Cell viability or proliferation was determined by using 3-(4, 5-dimethylthiazolyl-2-yl)-2–5 diphenyltetrazolium bromide (MTT). Cells were seeded in 96-well plates at 5 × 10^3^ per well in a final volume of 100 μl. After incubation for 2, 24, 48 and 72 hours, 10 μl (5 mg/ml) of MTT solution (Sigma Aldrich, St. Louis, USA) was added to each well and incubated for 4-hr at 37 °C. The supernatant was removed and 150 μl of DMSO was added. The absorbance of each sample was measured at 570 nm by a microplate spectrophotometer (Bio-Rad Laboratories, Hercules, CA, USA). Each experiment was performed in triplicate.

### Transwell assay

Invasion assays were performed at 37 °C for 24 hrs using 24-well transwell inserts (Corning Inc, Corning, NY, USA) coated with 20 μg (for MDA231 cells) or 12.5 μg (for MCF7 cells) matrigel (BD Biosciences, Franklin Lakes, NJ, USA). A total of 2 × 10^4^ freshly cultured cells in 200 μl serum-free medium were seeded into the upper chambers of the transwell filter, and the lower chambers were filled with 500 μl DMEM medium with 10% FBS as a chemoattractant. After incubation in a humidified atmosphere containing 5% CO_2_ at 37 °C, the matrigel filter was fixed with methanol and stained by 0.2% crystal violet. The cells on the upper chamber of the filters were removed by wiping with a cotton swab. The number of stained cells on the lower chamber of the filters was captured under the microscope at 200X magnification. Images of 10 randomly selected fields were counted using Image J software. Each experiment at least repeated for three times.

### Wound healing assay

Cells were seeded in 6-well tissue culture plates and grown to confluence in condition medium containing 2% FBS. Wounds were made by scraping with a sterilized 10 μl pipette tip, followed by extensive washing with phosphate-buffered saline to remove cellular debris. The migration distance was measured by the change in wound size during the 36–48 hours period. Photographs (magnification × 100) were taken at 0, 36, or 48 hours at the same position of the wound using an inverted microscope (Olympus, Tokyo, Japan). Where indicated, mitomycin-C (10 µM) was cultured with cells for 48 hours. The distance migrated by the cell monolayer to close the wounded area during the time period was measured using Image J software. Each experiment was repeated at least three times.

### Gelatin zymography

MMP-2 activity in culture medium was determined by gelatin zymography. Briefly, 1 × 10^6^ cells were seeded in 6-well tissue culture plates and cultured with serum-free medium for 24 hrs. Aliquots of conditioned supernatant (20 µl per well) was loaded onto 10% SDS polyacrylamide gels containing 0.1% gelatin. After electrophoresis, gels were washed in 2% (v/v) Triton X-100 to remove SDS, and then incubated in developing buffer [50 mM Tris-HCl (pH 7.4), 10 mM CaCl_2_ and 5 μM ZnCl_2_]. After 42 hrs incubation at 37 °C, the gels were stained for 4 hour using Coomassie Brilliant Blue R-250 and then destained extensively in 45% ethanol and 10% acetic acid. The protease activity was visualized as clear bands within the stained gel. The intensity of the bands was quantified using Image J software (NIH).

### Co-immunoprecipitation (Co-IP)

Nuclear extracts from cultured MDA231/TFPI-2 or MDA231/con cells were prepared. Co-IP experiments were performed using His•Bind Resin kit (Merck Millipore, Billerica, MA, USA) or protein A resins (Sigma Aldrich, USA) following the manufacturer’s instruction. The reciprocal co-IP was performed using AP-2α antibodies. Briefly, 20 µl of resins were incubated with 100 µl nuclear extracts (10 mg/ml) on ice for 4 hrs with gentle agitation. After centrifugation, the resins were washed three times with 500 µl of binding buffer, followed by two washes with 500 µl of wash buffer. Bound proteins were eluted with 30 µl of elute buffer. Eluted fractions were analyzed by western blots using anti-His, anti-TFPI-2 or anti-AP-2α antibodies.

### Immunofluorescence and Microscopy

Cells were cultured on glass coverslips, fixed with 4% paraformaldehyde solution for 30 minutes and treated with 0.1% Triton X-100 in PBS for 10 minutes. Cells were blocked with 1% BSA for 1 hr and incubated for 2 hrs at room temperature with primary antibodies. After washing three times with PBS, cells were incubated with Cy3- or FITC-conjugated secondary antibodies for an additional 1 h at room temperature in the dark. Finally, a drop of mounting medium containing DAPI was added and the cells were visualized under a confocal microscopy.

### Construction of MPP-2 Promoter-Luciferase Plasmids

The primers for generating promoter truncations of the *MMP-2* gene were listed in Supplemental Table 3. The PCR amplified promoter sequences were inserted into XhoI/EcoRI digested luciferase reporter plasmid pGL3 (Promega, Madison, WI, USA). All of the reporter cDNA constructs were purified using Qiagen DNA purification kits and were confirmed by restriction-enzyme-digestion analysis and sequencing.

### Transient Transfections and Luciferase Assays

TFPI-2 overexpression (MDA231/TFPI-2), TFPI-2 knockdown (MDA231/Sh2) and control (MDA231/con and MDA21/sh-con) cell clones were cultured in 6-well plates. When cells were 50–70% confluent, 5 μg of the reporter plasmid and 0.5 μg of pRL/CMV encoding a renella luciferase (internal control) were co-transfected into the cells by using the ProFection Mammalian Transfection System-DEAE-Dextran (Promega, Madison, USA). pGL3-basic plasmid was served as vector control. pRL-CMV plasmid was used to normalize the transfection efficiency. After 48 hrs transfection, the cells were rinsed with PBS and harvested. Luciferase assay was performed using the Dual-Luciferase Reporter Assay System (Promega, E1910) with a luminometer (Promega, USA). Luciferase activity was normalized by the internal control. Assays were performed in three independent experiments with duplicate transfection in each experiment.

### Chromatin immunoprecipitation (ChIP) assays

Magna ChIP A kit was purchased from MilliporeSigma (St. Louis, MO, USA). ChIP assays were performed using anti-TFPI-2, anti-AP-2α, anti-Histone H2 antibodies and control IgG based on the manufacturer’s instruction. Quantification of the immunoprecipitated DNA was performed by quantitative RT-time PCR in a S1000TM thermal cycler (Bio-Rad, Hercules, CA, USA) using SYBR Green PCR Core Reagents system (Applied Biosystems, Foster City, CA).

### Statistical analysis

The PASW Statistics 18 software package was used for statistical analysis. All numerical values reported represent means ± SD. Statistical significance compared with control values was calculated using the Student’s t-test (two tailed) and P < 0.05 indicates statistical significance. The statistical difference between more than two groups was evaluated by one-way ANOVA followed by Tukey’s multiple comparison test.

## Electronic supplementary material

Supplementary information

## References

[1] Sierko E, Wojtukiewicz MZ, Kisiel W (2007). The role of tissue factor pathway inhibitor-2 in cancer biology. Seminars in thrombosis and hemostasis.

[2] Iino M, Foster DC, Kisiel W (1998). Quantification and characterization of human endothelial cell-derived tissue factor pathway inhibitor-2. Arteriosclerosis, thrombosis, and vascular biology.

[3] Miyagi Y (1994). cDNA cloning and mRNA expression of a serine proteinase inhibitor secreted by cancer cells: identification as placental protein 5 and tissue factor pathway inhibitor-2. Journal of biochemistry.

[4] Chand HS (2004). The effect of human tissue factor pathway inhibitor-2 on the growth and metastasis of fibrosarcoma tumors in athymic mice. Blood.

[5] Herman MP (2001). Tissue factor pathway inhibitor-2 is a novel inhibitor of matrix metalloproteinases with implications for atherosclerosis. The Journal of clinical investigation.

[6] Kempaiah P, Chand HS, Kisiel W (2009). Human tissue factor pathway inhibitor-2 is internalized by cells and translocated to the nucleus by the importin system. Archives of biochemistry and biophysics.

[7] Xu Y (2011). Tissue factor pathway inhibitor-2 inhibits the growth and invasion of hepatocellular carcinoma cells and is inactivated in human hepatocellular carcinoma. Oncology letters.

[8] Vaitkiene P, Skiriute D, Skauminas K, Tamasauskas A (2012). Associations between TFPI-2 methylation and poor prognosis in glioblastomas. Medicina (Kaunas, Lithuania).

[9] Lavergne M (2013). Beneficial role of overexpression of TFPI-2 on tumour progression in human small cell lung cancer. FEBS open bio.

[10] Guo H (2007). Tissue factor pathway inhibitor-2 was repressed by CpG hypermethylation through inhibition of KLF6 binding in highly invasive breast cancer cells. BMC molecular biology.

[11] Rollin J (2005). Expression and methylation status of tissue factor pathway inhibitor-2 gene in non-small-cell lung cancer. British journal of cancer.

[12] Rao CN (2001). Expression of tissue factor pathway inhibitor 2 inversely correlates during the progression of human gliomas. Clinical cancer research: an official journal of the American Association for Cancer Research.

[13] Xu C (2013). Low expression of TFPI-2 associated with poor survival outcome in patients with breast cancer. BMC cancer.

[14] Zhai LL, Cai CY, Wu Y, Tang ZG (2015). Correlation and prognostic significance of MMP-2 and TFPI-2 differential expression in pancreatic carcinoma. International journal of clinical and experimental pathology.

[15] Izumi H, Takahashi C, Oh J, Noda M (2000). Tissue factor pathway inhibitor-2 suppresses the production of active matrix metalloproteinase-2 and is down-regulated in cells harboring activated ras oncogenes. FEBS letters.

[16] Gaud G (2011). TFPI-2 silencing increases tumour progression and promotes metalloproteinase 1 and 3 induction through tumour-stromal cell interactions. Journal of cellular and molecular medicine.

[17] Xu C (2012). The interaction of the second Kunitz-type domain (KD2) of TFPI-2 with a novel interaction partner, prosaposin, mediates the inhibition of the invasion and migration of human fibrosarcoma cells. The Biochemical journal.

[18] Kohrmann A, Kammerer U, Kapp M, Dietl J, Anacker J (2009). Expression of matrix metalloproteinases (MMPs) in primary human breast cancer and breast cancer cell lines: New findings and review of the literature. BMC cancer.

[19] Planque N (2006). Nuclear trafficking of secreted factors and cell-surface receptors: new pathways to regulate cell proliferation and differentiation, and involvement in cancers. Cell communication and signaling: CCS.

[20] Min KW (2016). NAG-1/GDF15 accumulates in the nucleus and modulates transcriptional regulation of the Smad pathway. Oncogene.

[21] Qin H, Sun Y, Benveniste EN (1999). The transcription factors Sp1, Sp3, and AP-2 are required for constitutive matrix metalloproteinase-2 gene expression in astroglioma cells. The Journal of biological chemistry.

[22] Sirover MA (2012). Subcellular dynamics of multifunctional protein regulation: mechanisms of GAPDH intracellular translocation. Journal of cellular biochemistry.

[23] Kuo TF, Tatsukawa H, Kojima S (2011). New insights into the functions and localization of nuclear transglutaminase 2. The FEBS journal.

[24] Rao CN (1998). HT-1080 fibrosarcoma cell matrix degradation and invasion are inhibited by the matrix-associated serine protease inhibitor TFPI-2/33 kDa MSPI. International journal of cancer. Journal international du cancer.

[25] Duffy MJ, Maguire TM, Hill A, McDermott E, O’Higgins N (2000). Metalloproteinases: role in breast carcinogenesis, invasion and metastasis. Breast cancer research: BCR.

[26] Marchant DJ (2014). A new transcriptional role for matrix metalloproteinase-12 in antiviral immunity. Nature medicine.

[27] Lee HK (2010). The odontogenic ameloblast-associated protein (ODAM) cooperates with RUNX2 and modulates enamel mineralization via regulation of MMP-20. Journal of cellular biochemistry.

[28] Nishibori M, Nakaya N, Ohtsuka A, Murakami T, Saeki K (1998). Localization of a serine proteinase inhibitor, B-43, in the bovine pancreas. Histochemistry and cell biology.

[29] Chuang TL, Schleef RR (1999). Identification of a nuclear targeting domain in the insertion between helices C and D in protease inhibitor-10. The Journal of biological chemistry.

[30] Bird CH (2001). Nucleocytoplasmic distribution of the ovalbumin serpin PI-9 requires a nonconventional nuclear import pathway and the export factor Crm1. Molecular and cellular biology.

[31] Strik MC (2002). Distribution of the human intracellular serpin protease inhibitor 8 in human tissues. The journal of histochemistry and cytochemistry: official journal of the Histochemistry Society.

[32] Scholtz B, Lamb K, Rosfjord E, Kingsley M, Rizzino A (1996). Appearance of nuclear protease activity after embryonal carcinoma cells undergo differentiation. Developmental biology.

[33] Orso F (2007). The AP-2alpha transcription factor regulates tumor cell migration and apoptosis. Advances in experimental medicine and biology.

[34] Song T (2015). Specific interaction of KIF11 with ZBP1 regulates the transport of beta-actin mRNA and cell motility. J Cell Sci.

