# Supplementary material for: Localization of TFPI-2 in the nucleus modulates MMP-2 gene expression in breast cancer cells
Source: Sci Rep. 2017 Oct 19;7:13575. doi: 10.1038/s41598-017-14148-8 (PMC5648759; doi:10.1038/s41598-017-14148-8)

SREP-17-16298C

Localization of TFPI-2 in the nucleus modulates MMP-2 gene expression in breast cancer cells

Guangli Wang[1§], Yao Zeng[1§], Shaoying Chen[1], Deling Li[1], Wei Li[1], Yanchun Zhou[1], Robert H. Singer2 and Wei Gu[1]*

Legends of Supplementary Information

Supplementary Figure 1 Role of TFPI-2 in proliferation and invasion of breast cancer cells

(A) Western blots indicate the expression of TFPI-2 in MDA231/Sh2 and shRNA control cells. (B) MTT assays were performed to measure the rate of cell proliferation in MDA231/Sh1, MDA231/Sh2 and shRNA control cells. Bars indicate standard error of mean from three independent experiments. *P<0.05. (C) Transwell assays indicated that, in comparison to the shRNA control cells, knockdown of TFPI-2 expression reduced invasive ability of MDA231 cells. The results are the average of three independent experiments each carried out in triplicate, ± SD. *P<0.05 as determined by one-way ANOVA followed by Tukey's multiple comparison tests. (D) Cell invasion assays indicated that re-expression of TFPI-2 in TFPI-2-knockdown cells inhibited cell invasion. The results are the average of three independent experiments each carried out in triplicate, ± SD. **P<0.01 as determined by Student's t-test. (E) Wound healing assays of MDA231 stable cell lines in the presence of mitomycin-C (10 μM). Wound closures were photographed at 48 hours after wounding. Data are statistically assessed from three independent experiments; ± SD. * P<0.05 as determined by one-way ANOVA followed by Tukey's multiple comparison tests. (F) Wound healing assays of MDA231/con cells in the absence or presence of mitomycin-C (10 μM). Wound closures were photographed at 48 hours after wounding. Data are statistically analyzed from three independent experiments; ± SD. * P<0.05 as determined by student's t-test.

Supplementary Figure 2 Expression of TFPI-2 in breast cancer cell lines

(A) qRT-PCR experiments showing the expression of TFPI-2 mRNA in MDA231, MCF7 and T47D cells. Data was analyzed from three independent experiments. *P<0.05 as determined by one-way ANOVA followed by Tukey's multiple comparison tests. (B) A representative Western Blot experiment indicates the expression of TFPI-2 protein in MDA231, MCF7 and T47D cells.

Supplementary Figure 3 Effect of exogenously offered TFPI-2 for cell proliferation and invasion

MCF7 cells were cultured in conditional medium harvested from MDA231/Sh2, MDA231/TFPI-2 and control cells. The effect of the treatments with conditional medium on proliferation, invasion and migration of MCF7 cells was analyzed using MTT tests (A), transwell assays (B) and wound healing assays (C), respectively. *P < 0.05; **P < 0.01 as determined by one-way ANOVA followed by Tukey's multiple comparison tests.

Supplementary Figure 4 TFPI-2 affects the MMP expression in breast cancer cells

(A) Western blots were performed to detect TFPI-2 expression in T47D/TFPI-2, T47D/con and T47D/WT cells using anti-TFPI-2 antibody. (B) qRT-PCR showing the relative levels of MMP-2 mRNAs in established stable T47D cell lines. Bars indicate standard error of mean from three independent experiments, *P<0.05. (C) and (D) qRT-PCR showing the relative levels of MMP-1 mRNA and MMP-9 mRNA in MDA231 cell lines. Bars indicate standard error of mean from three independent experiments.

Supplementary Figure 5 TFPI-2 affects the transcription of the MMP-2 promoter reporter

(A) Luciferase reporter driven by the -1030 to -1 bp of the MMP-2 promoter and an internal control Renilla luciferase plasmid were co-transfected into stable T47D cell lines. The firefly luciferase activity from each construct was normalized to the Renilla luciferase activity and the relative luciferase activity is represented. The results are the average of a total of three independent experiments each carried out in triplicate. *P<0.05. (B) Transwell assays indicated that knockdown of AP-2α reduced invasive ability of MDA231 cells. Data was analyzed from three independent experiments. **P<0.01 as determined by Student's t-test.

Supplementary Figure 6 Over-expression of MMP-2 increases invasive ability of MDA231/TFPI-2 cells

(A) A construct expressing MMP-2 protein was transfected into MDA231/TFPI-2 cells. Expression of MMP-2 in transfected cell lines was detected by western blots. (B) Transwell assays indicated that overexpression of MMP-2 increased invasive capability of MDA231/TFPI-2 cells. Bars indicate standard error of mean from three independent experiments. *P < 0.05, **P<0.01 as determined by one-way ANOVA followed by Tukey's multiple comparison tests.

Supplementary Figure 7 TFPI-2 affects the binding ability of Ap-2α to the MMP-2 promoter

ChIP assays were performed to examine the effect of TFPI-2 on the binding ability of AP-2α to the MMP2 gene promoter. The R1 and R2 are the two regions of the MMP-2 gene promoter as indicated on Fig. 6F. Protein A beads conjugated with AP-2α antibodies or IgG were used for ChIP assays. After co-immunoprecipitation, amplicons corresponding to R1 and R2 were amplified and analyzed. A representative agarose gel image indicated that AP-2α binds to the R1 region of the MMP-2 promoter and this binding was affected by over-expression of TFPI-2.

Supplementary tables

Table 1: Nucleotide sequences of shRNA specifically for knockdown of TFPI-2 in the study. Table 2: Primer sequences used for RT-PCR or quantitative RT-PCR to evaluate the expressions of TFPI-2, MMP-1, MMP-2, MMP-3, MMP-9, AP-2α and GAPDH transcripts in breast cancer cell lines. Table 3: Primers were designed to generate the cDNA fragments of the PPM-2 gene promoter by PCR. The locations of the primers on the MMP-2 promoter were indicated below the table.

Suppl. Fig.1

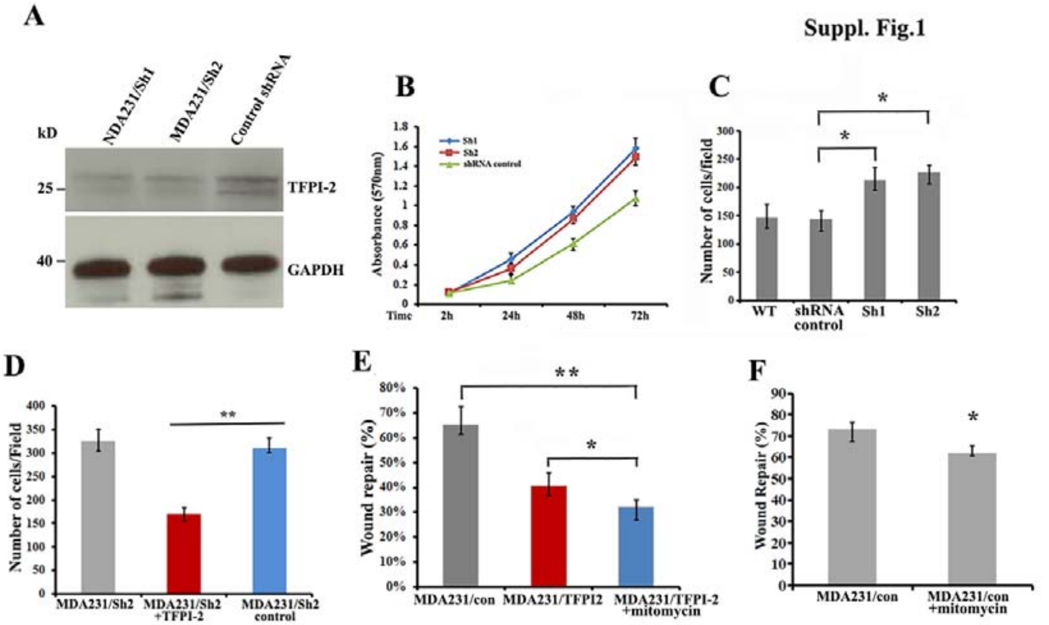

A
NDA231/Sh1
MDA231/Sh2
Control shRNA
kD
25
40
TFPI-2
GAPDH
B
Sh1
Sh2
shRNA control
Absorbance (570nm)
Time
2h
24h
48h
72h
C
Number of cells/field
WT
shRNA control
Sh1
Sh2
D
Number of cells/Field
MDA231/Sh2
MDA231/Sh2 +TFPI-2
MDA231/Sh2 control
E
Wound repair (%)
MDA231/con
MDA231/TFPI2
MDA231/TFPI-2 +mitomycin
F
Wound Repair (%)
MDA231/con
MDA231/con +mitomycin

Suppl. Fig.2

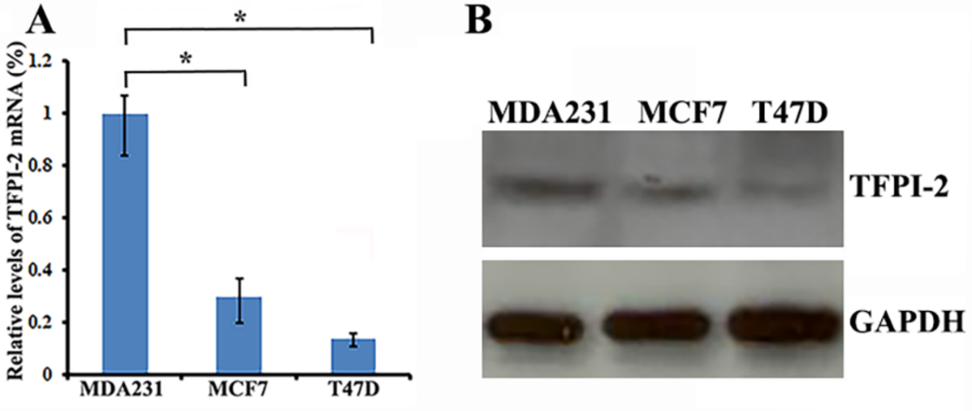
A
*
*
Relative levels of TFPI-2 mRNA (%)
1.2
1
0.8
0.6
0.4
0.2
0
MDA231
MCF7
T47D
B
MDA231
MCF7
T47D
TFPI-2
GAPDH

Suppl. Fig.3

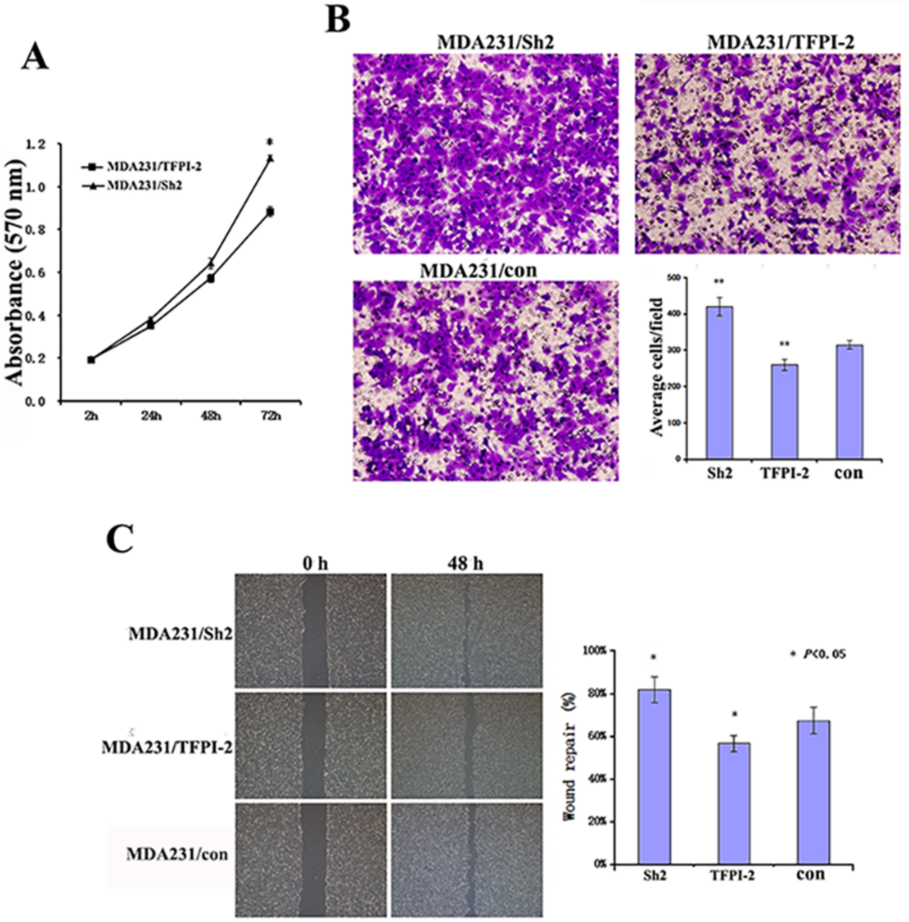
A
Absorbance (570 nm)
MDA231/TFPI-2
MDA231/Sh2
2h
24h
48h
72h
B
MDA231/Sh2
MDA231/TFPI-2
MDA231/con
Average cells/field
Sh2
TFPI-2
con
C
0 h
48 h
MDA231/Sh2
MDA231/TFPI-2
MDA231/con
Wound repair (%)
* P<0.05
Sh2
TFPI-2
con

Suppl. Fig. 4

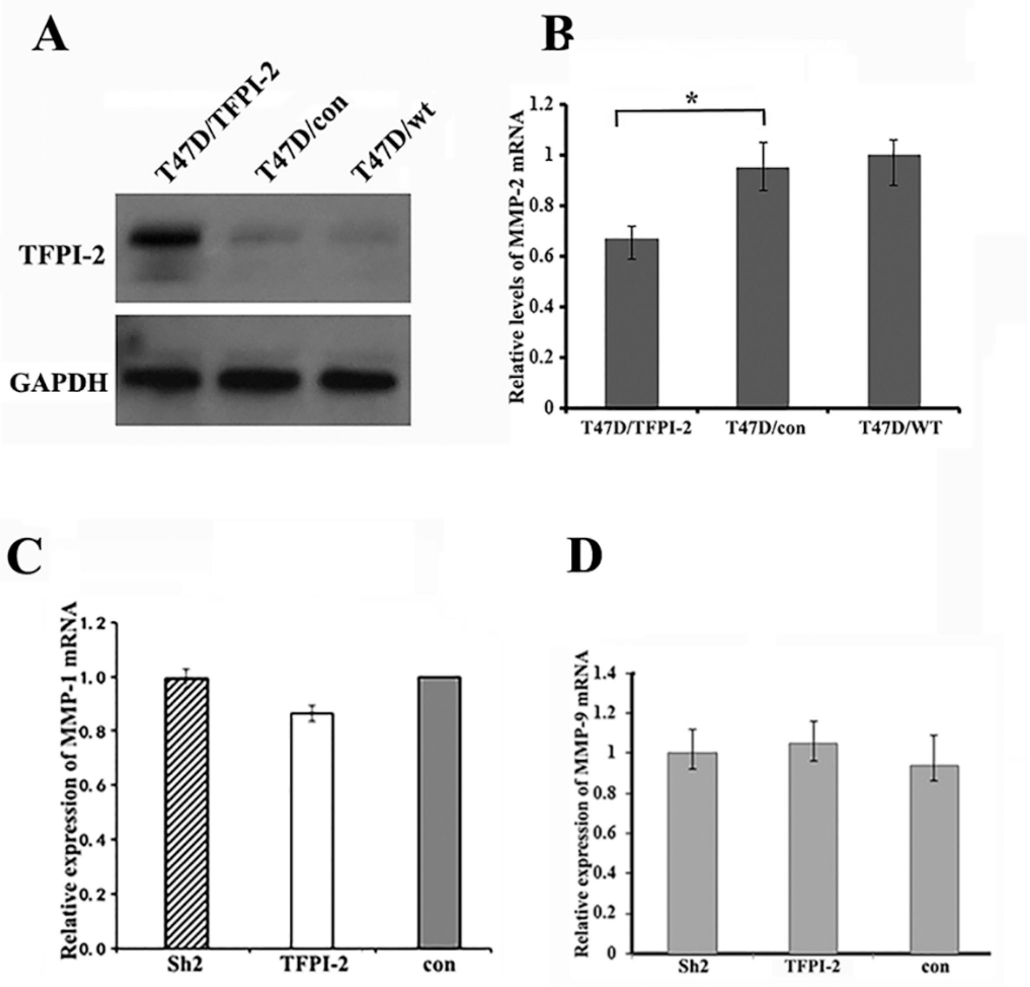
A
T47D/TFPI-2
T47D/con
T47D/wt
TFPI-2
GAPDH
B
Relative levels of MMP-2 mRNA
1.2
1
0.8
0.6
0.4
0.2
0
*
T47D/TFPI-2
T47D/con
T47D/WT
C
Relative expression of MMP-1 mRNA
1.2
1.0
0.8
0.6
0.4
0.2
0.0
Sh2
TFPI-2
con
D
Relative expression of MMP-9 mRNA
1.4
1.2
1
0.8
0.6
0.4
0.2
0
Sh2
TFPI-2
con

Suppl. Fig. 5

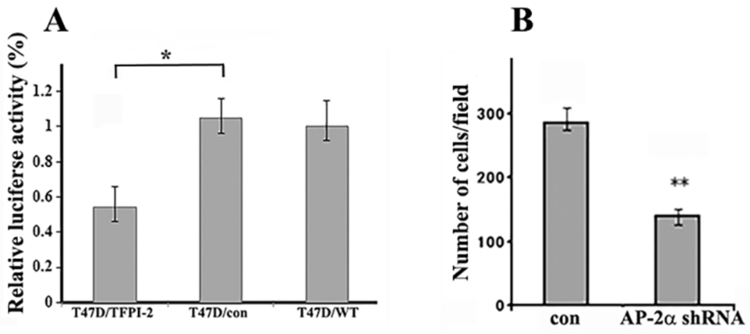
A
B
*
Relative luciferse activity (%)
0
0.2
0.4
0.6
0.8
1
1.2
T47D/TFPI-2
T47D/con
T47D/WT
Number of cells/field
0
100
200
300
**
con
AP-2α shRNA

Suppl. Fig. 6

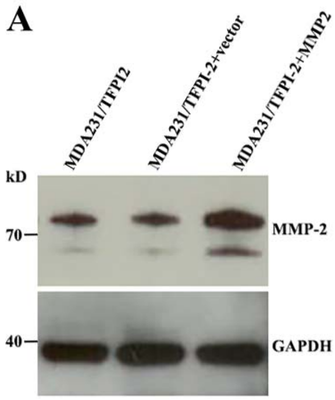

A
MDA231/TFPI2
MDA231/TFPI-2+vector
MDA231/TFPI-2+MMP2
kD
70
40
MMP-2
GAPDH

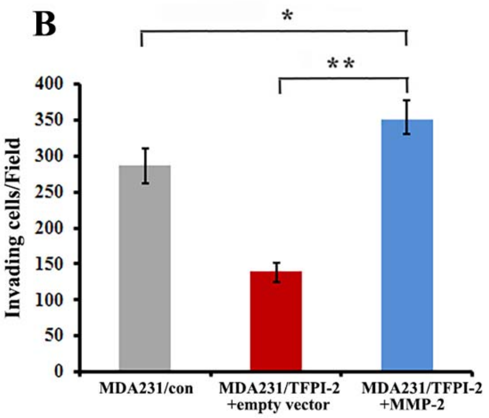

B
*
**
Invading cells/Field
400
350
300
250
200
150
100
50
0
MDA231/con
MDA231/TFPI-2 +empty vector
MDA231/TFPI-2 +MMP-2

Suppl. Fig.7

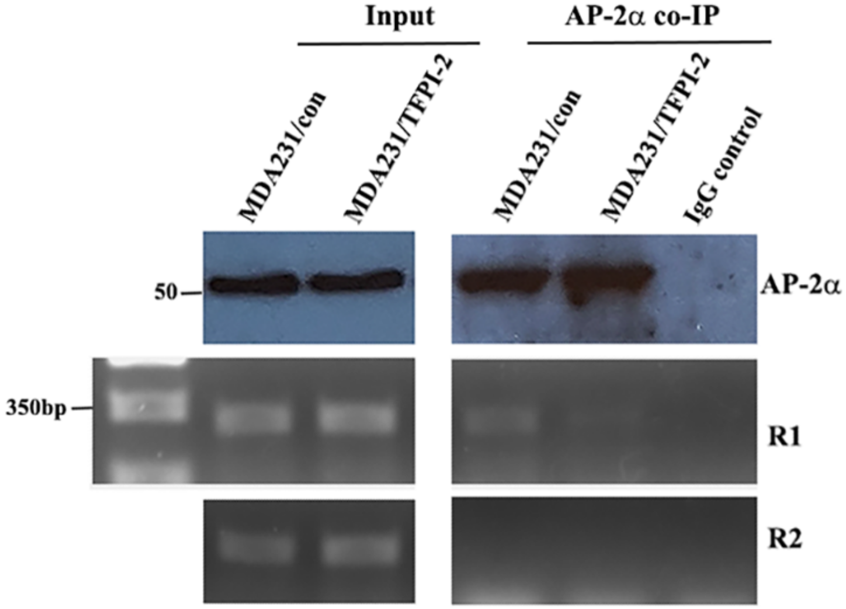
Input
AP-2α co-IP
MDA231/con
MDA231/TFPI-2
MDA231/con
MDA231/TFPI-2
IgG control
50
AP-2α
350bp
R1
R2

Table 1. shRNAs specifically for TFPI-2 knockdown

Name	Strand	Sequences (5'-3')
shRNA-1	top	tcgagttgcgacgatgcttgctggaggatagaaatcaagagtttctatcctccagcaagcatcgtcgcaattttttg
	bottom	aattcaaaaaattgcgacgatgcttgctggaggatagaaactcttgatttctatcctccagcaagcatcgtcgcaac
shRNA-2	top	tcgagccaagatacagaacctgtgatgctttcactcaagaggtgaaagcatcacaggttctgtatcttggttttttg
	bottom	aattcaaaaaaccaagatacagaacctgtgatgctttcacctcttgagtgaaagcatcacaggttctgtatcttggc

Table 2. Primers used for RT-PCR and qRT-PCR experiments

Gene	Forward primers	Reverse primers
TFPI-2	GTCCCAAGAAGACAAAGTCGCA	GTGGTCTCCAACCCACAATGTC
MMP-1	CTGCTGCTGTTCTGGGGT	GCCACTATTTCTCCGCTTTTC
MMP-2	AGCTCCCGGAAAAGATTGATG	CAGGGTGCTGGCTGAGTAGAT
MMP-3	ATCCCGAAGTGGAGGAAAAC	GCCTGGAGAATGTGAGTGGA
MMP-9	CCTGGAGACCTGAGAACCAATC	GATTTCGACTCTCCACGCATCT
AP-2α	GTTACCCTGCTCACATCACTAG	TCTTGTCACTTGCTCATTGGG
GAPDH	GAGTCAACGGATTTGGTCGT	TGGGATTTCCATTGATGACA
MMP-2 promoter R1	GTGGCTGATCATCTGTTTCTGACC	CTACTCCTGGCCTCTACGTC
MMP-2 promoter R1	CAAGAGTGAGTGGGGAATTCGTGG	AACAGTATGCAGTGAAGAAGCCAG

Table 3. Primers used for amplifying MMP-2 promoter

Name	Positions	Sequences (5’-3’)
MMP2-F1	-1030	CAAGAGTGAGTGGGGAATTCGT
MMP2-R1	-1	TGCAGCGGAAACAAGGGAGGG
MMP2-F2	-650	CTATACGAGGCCAAGTTAAGGC
MMP2-F3	-310	GCTGCTCTCTAACCTCAGGACG
MMP2-R2	-310	TGCGAGATGCTAGATACACCTT

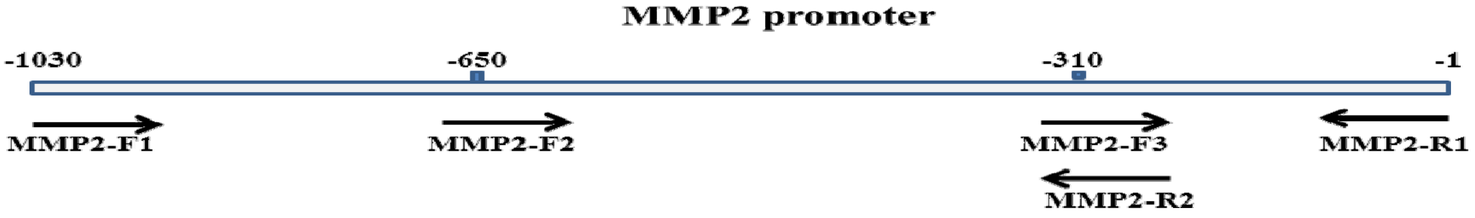

Supplement: Supplementary file 1 — Supplementary information [file 41598_2017_14148_MOESM1_ESM.pdf]
